# Premedication in pediatric Meckel scintigraphy: pantoprazole versus ranitidine for optimizing scan quality

**DOI:** 10.1007/s00247-025-06284-5

**Published:** 2025-06-14

**Authors:** Hasan Önner, Merve Ni̇da Calderon Tobar, Mehmet Sarıkaya, Fatma Özcan Sıkı, Fari̇se Yılmaz, Muslu Kazim Körez, Gonca Kara Gedi̇k

**Affiliations:** 1 Selçuk University, Medical Faculty, Department of Nuclear Medicine, Konya, Türkiye; 2 Selçuk University, Medical Faculty, Department of Pediatric Surgery, Konya, Türkiye; 3 Selçuk University, Medical Faculty, Department of Biostatistics, Konya, Türkiye

**Keywords:** Ectopic gastric mucosa, Meckel diverticulum, Pantoprazole, Pediatric, Ranitidine, Scintigraphy

## Abstract

**Background:**

The standard method for diagnosing Meckel diverticulum and identifying ectopic gastric mucosa is ^99 m^Tc-pertechnetate imaging. Premedication with H_2_ receptor antagonists enhances the scan’s sensitivity by reducing the washout of ^99 m^Tc-pertechnetate activity from the intestinal lumen.

**Objective:**

After the withdrawal of ranitidine, we compared the efficacy of the proton pump inhibitor pantoprazole as an alternative premedication agent for ^99 m^Tc-pertechnetate Meckel diverticulum imaging.

**Materials and methods:**

This study assessed the scan quality of 141 children (aged 1 month to 204 months (median = 84 months)) who underwent Meckel scintigraphy over 10 years at a single institution. Before its withdrawal in December 2020, ranitidine was utilized for premedication, while pantoprazole was used thereafter. Therefore, patients were divided into two premedication groups: ranitidine (*n* = 88) and pantoprazole (*n* = 53). A high-quality scan was defined as having no ^99 m^Tc-pertechnetate activity in the duodenal and other intestinal lumens. The effectiveness of pantoprazole in reducing ^99 m^Tc-pertechnetate release in the duodenum and other intestinal lumens was compared to that of ranitidine. Differences in scan quality between the groups were analyzed using the two-proportion *Z*-test. In patients with positive scans, the lesion-to-background activity ratio of the Meckel diverticulum was measured and compared between the premedication groups.

**Results:**

Premedication with pantoprazole resulted in 47.2% of scans showing no ^99 m^Tc-pertechnetate release, 37.7% with activity localized either in the duodenum or other intestine, and 15.1% exhibiting activity in both regions. In comparison, ranitidine resulted in 45.5% of scans with no ^99 m^Tc-pertechnetate release, 40.9% with activity localized either in the duodenum or other intestine, and 13.6% showing activity in both regions. *P*-values were not found to be significant in all comparisons. Twelve scans were positive; all patients had Meckel diverticulum confirmed at surgery. For positive scans, the lesion-to-background activity ratio for the Meckel diverticulum was similar between the ranitidine and pantoprazole groups.

**Conclusion:**

This study demonstrates that pantoprazole is statistically non-inferior to ranitidine regarding scan quality and lesion-to-background activity ratios for Meckel diverticulum detection. Pantoprazole offers a reliable alternative for clinical protocols in the absence of ranitidine.

**Graphical Abstract:**

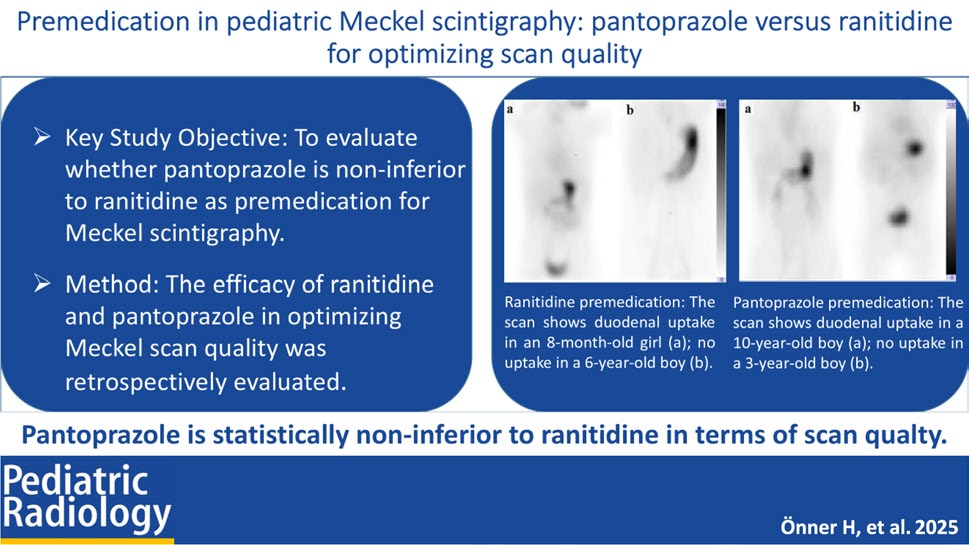

## Introduction

Johann Meckel first described the Meckel diverticulum in 1812 as a vestigial remnant of the omphalomesenteric duct [1]. It is the most common gastrointestinal congenital anomaly, occurring in 2–3% [2, 3].

Diagnosis typically relies on ^99 m^Tc-pertechnetate scintigraphy (Meckel diverticulum scintigraphy), which is highly sensitive in detecting ectopic gastric mucosa [3, 4]. However, the scan’s diagnostic accuracy can be influenced by various factors, including the patient’s preparation before imaging [4]. Premedications commonly include H_2_ receptor blockers (famotidine, cimetidine, and ranitidine), pentagastrin, and glucagon [5, 6]. However, pentagastrin and glucagon are less favored due to their side effects [5].

Traditionally, H_2_ receptor antagonists such as ranitidine improved scanning quality by minimizing the washout of ^99 m^Tc-pertechnetate from the gastric mucosa into the intestinal lumen. This approach enhances radiotracer retention at sites of ectopic gastric mucosa, improving imaging contrast and sensitivity [6]. The Food and Drug Administration banned ranitidine after detecting N-nitrosodimethylamine, a potential carcinogen linked to lung, liver, and bladder cancer in animal studies [7]. This led to its global withdrawal from medical markets [8], driving the search for alternative premedication agents.

Proton pump inhibitors represent a potential substitute given their ability to suppress gastric acid secretion more effectively and for longer than H_2_ receptor blockers. Proton pump inhibitors are well-suited to replace H_2_ inhibitors due to their rapid onset and advantageous pharmacokinetics following intravenous administration [9]. Despite their widespread use in clinical practice for acid suppression, limited data exist on their efficacy in Meckel diverticulum scintigraphy [10, 11].

This study aims to evaluate the effectiveness of pantoprazole as a premedication agent and to compare its performance with the established ranitidine protocol. Pantoprazole was chosen based on its well-documented safety profile in pediatric populations, favorable pharmacokinetic characteristics—particularly its minimal interaction with cytochrome P450 enzymes such as CYP2 C19 and CYP3 A4—and its routine use in clinical practice at our institution. By addressing this gap, we aim to identify a practical and effective alternative for optimizing image quality in Meckel scintigraphy.

## Materials and methods

This retrospective study analyzed data from 141 children who underwent Meckel diverticulum scintigraphy for suspected Meckel diverticulum at a single institution between January 2012 and December 2024.

Inclusion criteria included patients with clinical suspicion of ectopic gastric mucosa and the availability of complete imaging and premedication records. Patients with incomplete data or alternative diagnoses were excluded. All positive scans were confirmed histopathologically after surgical resection. The Local Research Ethics Committee approved the study (893,157/2025), and the requirement for written consent was waived. This study was performed in accordance with the ethical standards laid down in an appropriate version of the 1964 Declaration of Helsinki.

Meckel diverticulum scintigraphy was conducted following the practice guidelines [5]. A 4-h fasting period was ensured before the scan. After the market withdrawal of H_2_ receptor antagonists in December 2020, our institution implemented intravenous pantoprazole as the standard premedication. Premedications were given 30 min before the scan: intravenous ranitidine (1 mg/kg; max 50 mg) from 2012 to 2020 and intravenous pantoprazole (20 mg for those under 10 years, 40 mg for those over 10 years) from 2021 to 2024. The radiotracer (1.85 MBq/kg with a minimum of 18.5 MBq) was administered intravenously [12]. Dynamic images of the anterior abdomen were acquired at a frame rate of 1 image every 30 s for at least 30 min using a gamma camera (E CAM, Siemens Healthineers, Hoffman Estates, IL) equipped with a low-energy, high-resolution collimator. Additional static images—including anterior, anterior oblique, lateral, and posterior projections—were obtained after dynamic acquisition. Lateral views were used to localize renal pelvic activity, while postvoid images aided in detecting Meckel diverticulum activity potentially obscured by the urinary bladder. In cases where voluntary voiding was not possible, bladder emptying was achieved using a urinary catheter.

Scan quality was independently evaluated by H.Ö., a nuclear medicine physician with 8 years of experience, and M.N.C.T., a nuclear medicine resident with 3 years of training. Each scan was assessed for ^99 m^Tc-pertechnetate activity within the duodenum or other intestinal lumens, or both. Scan quality was rated based on visualization of intestinal activity (absent, duodenal, or other intestinal lumens), as previously defined [11]. High-quality scans were defined by the absence of radiotracer activity in the duodenum or other intestinal lumens. A focal area of radiotracer uptake that appears simultaneously with gastric mucosal activity in the abdomen is regarded as a positive Meckel scan, indicating ectopic gastric mucosa. Any interobserver discrepancies were resolved by consensus.

The lesion-to-background activity ratio of the Meckel diverticulum was measured in positive scans. For background activity measurement, a spherical region of interest (ROI) of the same size as the ROI drawn for Meckel’s diverticulum was drawn from the left lower quadrant, where no activity was observed.

### Statistical analysis

The scan quality following pantoprazole premedication was compared to that after ranitidine administration. Data were analyzed using descriptive statistics and chi-square tests to compare the proportions of high-quality scans between premedication groups. Scan quality differences between groups were evaluated using the two-proportion *Z*-test. In addition, the lesion-to-background activity ratio in positive scans in both premedication groups was compared with the Mann–Whitney *U* test. The *P*-value of < 0.05 was considered statistically significant.

## Results

From January 2012 to December 2024, 148 patients underwent a Meckel scan for lower-intestine hemorrhage etiology. Scans without information about the premedication procedure were excluded (*n* = 7). Among the 141 children included, ages ranged from 1 to 204 months (median = 84 months), and 72 (51%) were male. Ranitidine was given to 88 patients (62%), while 53 (38%) received pantoprazole for premedication. The characteristics of the patients are shown in Table 1.

**Table 1:** The characteristics of patients

Age median (range months)	84 (1–204)
Sex, *n* (%)	
Male	72 (51%)
Female	69 (49%)
Premedication, *n* (%)	
Ranitidine	88 (62%)
Pantoprazole	53 (38%)
Scintigraphy results, *n* (%)	
Positive	12 (8.1%)
Negative	129 (91.9%)

**Fig. 1:**
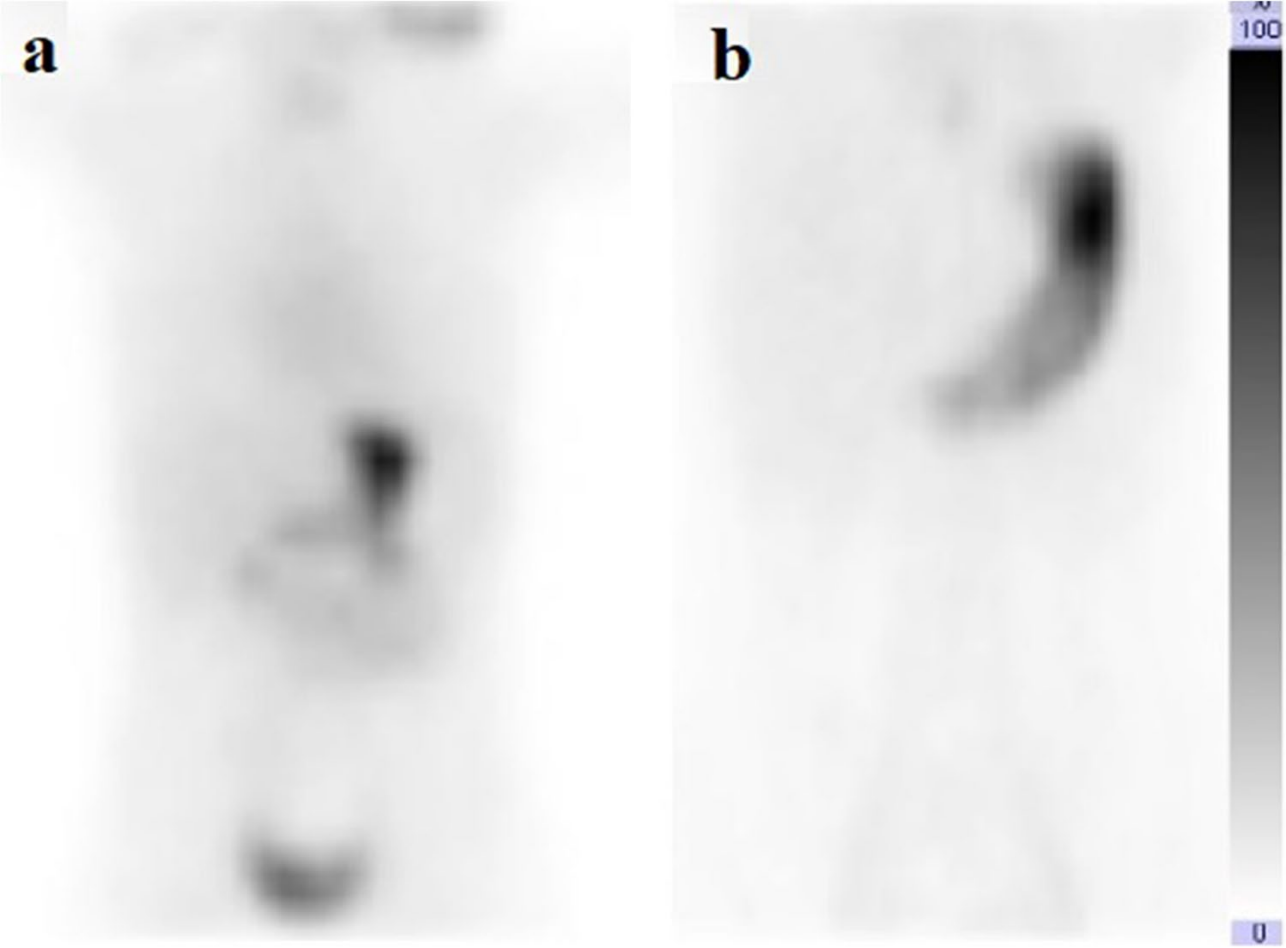
Coronal whole-body.^99 m^Tc-pertechnetate scans after ranitidine premedication demonstrate differing findings in two pediatric patients evaluated for suspected Meckel’s diverticulum as the cause of lower gastrointestinal bleeding. Focal tracer accumulation is observed in the duodenum and other intestinal lumens in an 8-month-old girl (**a**), whereas no visible tracer uptake is seen in the duodenum or intestinal lumen in a 6-year-old boy (**b**)

**Fig. 2:**
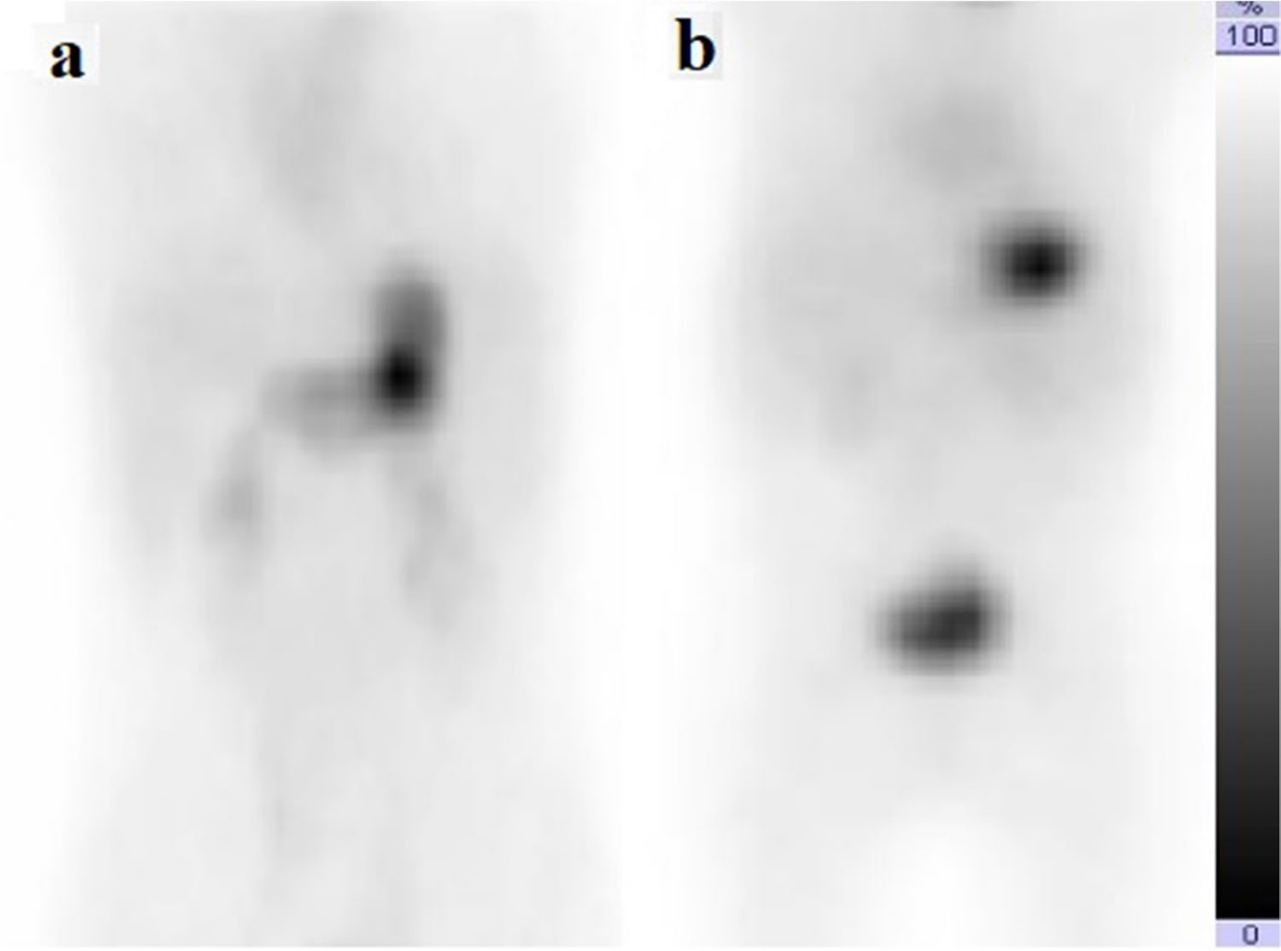
Coronal whole-body.^99 m^Tc-pertechnetate scans following pantoprazole premedication in two pediatric patients evaluated for suspected Meckel’s diverticulum as the cause of lower gastrointestinal bleeding. Focal tracer accumulation is seen in the duodenum of a 10-year-old boy (**a**), while no visible uptake is observed in the duodenum or other intestinal lumens in a 3-year-old boy (**b**)

Each scan was evaluated for the presence or absence of activity in the duodenal lumen or other intestinal lumens, or both. Premedication with pantoprazole led to 47.2% of scans showing no ^99 m^Tc-pertechnetate release, 37.7% displaying activity localized to either the other intestine or duodenum, and 15.1% exhibiting activity in both regions. Ranitidine resulted in 45.5% of scans without ^99 m^Tc-pertechnetate release, 40.9% with activity confined to the other intestine or duodenum, and 13.6% with activity in both areas. No significant differences were found in any comparisons (*P* > 0.05). Table 2 presents the comparison of Meckel diverticulum scintigraphy quality between premedication groups. Figures 1 and 2 illustrate the anterior planar images after premedication with ranitidine and pantoprazole, respectively.

**Table 2:** Comparison of Meckel scintigraphy quality between premedication groups

	Ranitidine (*n* = 88)	Pantoprazole (*n* = 53)	Difference [95% CI]	*P*-value
Absent in duodenal lumen, *n* = 97	63 (71.6%)	34 (64.2%)	7.44 [7.94 to 23.28]	0.357
Absent in other intestinal lumens, *n* = 89	53 (60.2%)	36 (67.9%)	7.69 [8.79 to 22.84]	0.361
Absent in duodenal and other intestinal lumens, *n* = 65	40 (45.5%)	25 (47.2%)	1.72 [14.74 to 18.25]	0.843
Activity in duodenal or other intestinal lumens, *n* = 56	36 (40.9%)	20 (37.7%)	3.17 [13.40 to 18.92]	0.710
Activity in both duodenal and other intestinal lumens, *n* = 20	12 (13.6%)	8 (15.1%)	1.45 [9.87 to 14.68]	0.811

*CI*, confidence interval

The prevalence of Meckel diverticulum in our population was 8.1%. All 12 children with positive scans had histopathologically confirmed Meckel diverticulum with the presence of ectopic gastric tissue. The lesion-to-background activity ratio in positive scans was comparable between the ranitidine (*n* = 7; median = 2.58) and pantoprazole (*n* = 5; median = 2.36) premedication groups (*P* > 0.05).

## Discussion

Following the 2020 market withdrawal of H_2_ receptor antagonists, our institution adopted intravenous pantoprazole as the standard premedication due to its rapid onset of action, excellent pharmacokinetic profile, and robust evidence supporting its efficacy and safety in the pediatric population [9]. The results of this study suggest that pantoprazole provides diagnostic performance equivalent to ranitidine for imaging Meckel diverticulum, without compromising scan quality or lesion-to-background activity ratios.

A direct comparison was not possible due to the lack of availability of ranitidine; therefore, retrospective analysis of available data was used in this study as the most appropriate alternative. H_2_ receptor antagonists enhance the sensitivity of Meckel scintigraphy by suppressing gastric acid secretion—though the precise mechanism remains unclear—and by delaying the luminal release of ^99 m^Tc-pertechnetate from the parietal and mucus cells, thereby increasing radiotracer concentration in both the stomach and Meckel diverticulum [6, 13, 14]. Pantoprazole, an irreversible proton pump (H⁺/K⁺-ATPase) inhibitor, similarly reduces acid secretion from gastric parietal cells and is thought to augment ^99 m^Tc-pertechnetate uptake via a comparable mechanism [15]. In the preclinical study by Gültekin et al., pantoprazole premedication was shown to increase ^99 m^Tc-pertechnetate uptake in the gastric wall of rabbits, supporting its potential role as a premedication agent in Meckel diverticulum scintigraphy [10].

In the study by Ververs et al., which also informed our methodological approach, esomeprazole premedication was shown to be more effective than ranitidine in suppressing the gastrointestinal release of ^99 m^Tc-pertechnetate during Meckel diverticulum scintigraphy [11]. In contrast, our findings demonstrated that pantoprazole premedication was equivalent to ranitidine, with no significant difference observed between the two agents. Several pharmacological factors may account for this discrepancy. Although pantoprazole and esomeprazole are proton pump inhibitors that irreversibly inhibit the H⁺/K⁺-ATPase enzyme in gastric parietal cells to reduce acid secretion [16], they differ in key pharmacokinetic properties. Pantoprazole is a racemic mixture, while esomeprazole is the S-enantiomer of omeprazole and exhibits more consistent bioavailability and acid suppression [17]. Furthermore, esomeprazole’s stronger dependence on CYP2 C19 metabolism may contribute to variable individual responses [18], whereas pantoprazole is less affected by genetic polymorphisms, leading to more predictable pharmacokinetics [19]. Some studies suggest esomeprazole may achieve slightly more significant and prolonged acid suppression than pantoprazole, particularly at standard dosing [20, 21].

The prevalence of Meckel diverticulum in our population (8.1%) is consistent with earlier studies [11, 22], with a corresponding specificity of 100% for diagnosing ectopic gastric tissue. Radiologic evaluation of Meckel diverticulum varies depending on the clinical presentation and complications. Fluoroscopic small bowel enemas have been used historically [23]. Ultrasound is generally of limited diagnostic value but may occasionally demonstrate a blind-ending, peristaltic loop connected to the small bowel [24, 25]. Computed tomography (CT) is similarly limited in uncomplicated presentations, as the diverticulum can resemble a normal bowel loop. Nevertheless, CT may reveal a fluid- or air-filled, blind-ending pouch arising from the antimesenteric border of the distal ileum or, in rare cases, an inverted diverticulum appearing as an intraluminal polypoid mass [25, 26]. In cases of gastrointestinal bleeding, angiography may detect a persistent omphalomesenteric artery; however, overlying vasculature often hinders identification. Among the imaging modalities, ^99 m^Tc-pertechnetate scintigraphy remains the most useful for detecting ectopic gastric mucosa within the diverticulum.

This study has several limitations. First, the unavailability of ranitidine precluded a direct head-to-head comparison with pantoprazole, limiting the ability to evaluate relative efficacy. Second, the retrospective design and small sample size may introduce bias and limit the generalizability of the findings. Additionally, due to the limited number of patients in the subgroups, propensity score matching could not be performed. However, all patients were consecutively included to minimize selection bias.

## Conclusion

Based on this study’s findings, pantoprazole may serve as an effective alternative to ranitidine for Meckel diverticulum, demonstrating comparable diagnostic performance without compromising scan quality or lesion-to-background activity ratios. However, larger prospective studies are warranted to confirm these results and further validate the clinical utility of pantoprazole in this setting.

## Data Availability

The data that support the findings of this study are available from the corresponding author upon reasonable request.
